# Evaluation of Disease Spectrum in Hospitalized Cats with Hyperlipasemia: Pancreatitis Alone Is Rare, Hyperlipasemia without Suspected Pancreatitis Is Common

**DOI:** 10.3390/ani14101479

**Published:** 2024-05-16

**Authors:** Vanessa Hotz, Daniel Brugger, Peter Hendrik Kook

**Affiliations:** 1 Clinic for Small Animal Internal Medicine, Vetsuisse Faculty, University of Zurich, 8057 Zurich, Switzerland; vanessa.hotz@uzh.ch; 2 Institute of Animal Nutrition and Dietetics, Vetsuisse Faculty, University of Zurich, 8057 Zurich, Switzerland; dbrugger@nutrivet.uzh.ch

**Keywords:** cats, hospitalization, pancreatitis, pancreatopathy, lipase activity, fPLI, concurrent disease, comorbidity, ultrasonography

## Abstract

**Simple Summary:**

Pancreatitis is a common histological finding in cats, but it is unknown how often cats receive a clinical diagnosis of pancreatitis. Diagnosing pancreatitis in cats is challenging because of nonspecific clinical signs and a lack of diagnostic lipase cutoffs. It is particularly unclear how often clinicopathological evidence of pancreatitis in the presence of extra-pancreatic disease does not lead to a diagnosis of pancreatitis. Thus, we retrospectively evaluated the extent of comorbidities in 563 hospitalized cats with hyperlipasemia. Medical records were searched, and all available diagnoses were recorded and grouped by organ system. Medical findings were compared between cats with pancreatitis alone (PA), pancreatitis with concurrent disease (PD), and no suspected pancreatitis (NP). PA was rare (33 cats (6%)), 159 cats (28%) were in the PD group, and 371 cats (66%) had NP. Clinical, laboratory, and ultrasonographic findings were not different between PA and PD cats. Lipase activities did not differ between the three groups. Common disease categories in PD and NP cats were gastrointestinal, hepatobiliary, renal/urinary, and endocrine, and renal/urinary, gastrointestinal, cardiac, and musculoskeletal, respectively. In summary, cats are rarely hospitalized only because of suspected pancreatitis and the majority of cats have comorbidities, but clinical differences were not found between the PA and PD groups. Future studies are necessary to assess if increased lipase in sick cats with primary non-pancreatic disease stems from secondary pancreatic injury or is due to preexisting chronic pancreatitis.

**Abstract:**

Histological evidence of pancreatitis is commonly found in necropsy studies in cats. A clinical diagnosis of pancreatitis is challenging due to nonspecific clinical signs, a lack of diagnostic lipase cutoffs, and frequent presence of multiple diseases. It is still unknown how often pancreatitis alone is found in sick cats and how often clinicopathological evidence of pancreatitis in sick cats does not lead to a clinical diagnosis of pancreatitis. Our aims were to evaluate the extent of comorbidities in cats with suspected pancreatitis, evaluate how often sick cats with hyperlipasemia are diagnosed only with non-pancreatic diseases, and compare their clinical findings. Medical records of 563 client-owned hospitalized cats with available lipase activity measurement (LIPC Roche) > 30 U/L (RI, 6–26) were searched and medical diagnoses recorded and grouped by organ system. Clinicopathological findings were compared between cats with pancreatitis alone (PA), pancreatitis with concurrent disease (PD), and no suspected pancreatitis (NP). We found that PA was present in 33 (6%) cats, 159 cats (28%) were in the PD group, and 371 cats (66%) had no suspected pancreatitis (NP). Clinical, laboratory, and ultrasonographic findings did not differ between PA and PD cats. Lipase activities did not differ between the three groups. The most common disease categories in PD and NP cats were gastrointestinal, hepatobiliary, renal/urinary, and endocrine, and renal/urinary, gastrointestinal, cardiac, and musculoskeletal, respectively. We conclude that cats are rarely hospitalized because of suspected pancreatitis alone, and PA cats did not differ clinically from PD cats. Hyperlipasemia in sick cats without a diagnosis of pancreatitis may be due to a reactive pancreatopathy or preexisting chronic pancreatitis.

## 1. Introduction

Pancreatitis is frequently found histopathologically in necropsy studies in cats [1,2]. The gold standard for diagnosing pancreatitis is biopsy, but because pancreatic biopsies are invasive, lack therapeutic consequences, and may still miss localized lesions, pancreatitis is almost always a clinical diagnosis based on clinical, laboratory, and ultrasonographic (US) findings [3,4]. The fact that clinical signs in cats with pancreatitis are usually vague and unspecific and do not differ between acute and chronic forms either [5] hampers a first clinical preselection for more specific diagnostics. Determination of serum lipase is widely used for a clinical pancreatitis diagnosis [6]. Lipase is mostly measured using an immunoassay (pancreatic lipase immunoreactivity measuring species-specific pancreatic lipase, fPLI) or a catalytic assay using the substrate 1,2-o-dilauryl-rac-glycero-3-glutaric acid-(6′-methylresorufin)-ester (DGGR) [2,7,8,9]. Both assays have repeatedly been shown to correlate highly [2,9,10,11], and performed equally when compared to a standardized histological examination of the entire pancreas or pancreatic US assessment [2,9]. Similar to dogs, there is also now increasing discussion that in cats, that the pancreas could be affected secondarily by hemodynamic compromise stemming from a primary non-pancreatic disease [12,13,14,15]. While comorbidities such as gastrointestinal, hepatobiliary, and renal disease in cats with pancreatitis have been often documented histologically [5,16,17,18,19,20], clinical information is limited [12,21,22]. In particular, it remains unknown how often pancreatitis is the only disease found in sick cats and whether cats with pancreatitis alone differ clinically from cats with pancreatitis and comorbidities. At our hospital, DGGR-lipase activity (LIPC Roche) is included in the routine chemistry profile, and no extra request is needed when testing for pancreatitis compared to institutions using fPLI. This means lipase results are also available in cases where non-pancreatic disease already explains all clinical findings. This situation allowed us to retrospectively investigate the disease spectrum in a large number of cats with increased lipase activity.

The aims of our study were threefold: firstly, to examine how often hospitalized cats with increased lipase activity receive a clinical diagnosis of pancreatitis; secondly, to assess the extent of comorbidities in cats with suspected pancreatitis; and thirdly, to assess how often sick cats with increased lipase are diagnosed with only non-pancreatic disease. To better characterize the different groups, we also compared clinical, laboratory, and US findings between groups. We hypothesized that pancreatitis alone would be rare and that most cats with pancreatitis would have at least one additional disease. Because of the nonspecific clinical signs in cats with pancreatitis and the well-known poor agreement of lipase with US evidence of pancreatitis [9,23,24,25], we further hypothesized that many cats with non-pancreatic diseases would not receive a clinical diagnosis of pancreatitis despite increased lipase values.

## 2. Materials and Methods

### 2.1. Case Selection and Data Collection

Cases were identified by searching medical records at the authors’ institution between October 2017 and January 2020. Inclusion criteria were hospitalization and inpatient treatment for at least 24 h and a lipase activity result > 30 U/L (RI, 6–26 U/L) [8]. When a cat met these criteria more than once, only data from the first hospitalization were recorded. Data collection encompassed signalment, clinical, laboratory (CBC, chemistry), and US findings at presentation, as well as final diagnoses. Clinical signs were obtained from medical history and initial examination reports, focusing on the presence of vomiting, diarrhea, lethargy, anorexia, painful abdomen, polyuria, polydipsia (Pu/Pd), and weight loss. Medical diagnoses were taken from referral letters and medical summaries and were categorized by organ system. Cats with a diagnosis of pancreatitis alone without additionally recorded diagnoses were grouped in the clinical diagnosis pancreatitis alone (PA) group. Cats with a diagnosis of pancreatitis that had at least one additional comorbidity were grouped into the pancreatitis and concurrent disease (PD) group. The third group encompassed all cats without a clinical pancreatitis diagnosis, hereafter referred to as no pancreatitis (NP). All comorbidities from cats in the PD and NP groups were categorized into the following groups: gastrointestinal disease, renal/urinary disease, hepatobiliary disease, endocrine disease, cardiac disease, respiratory disease, musculoskeletal disease, neurological disease, hematological immune-mediated disease, ophthalmologic disease, and neoplastic disease. Duration of clinical signs before presentation and duration of hospitalization were also recorded. Cases were seen by interns, residents, and staff clinicians, and all cases were supervised by board-certified internists.

To compare pancreatic US findings and concurrent hepatobiliary and gastrointestinal US findings frequently associated with pancreatitis, presence of pancreatic, hepatobiliary, and gastrointestinal US abnormalities was also recorded. All US findings were recorded separately from the medical diagnoses. It is important to note that, as with all other clinical findings, US findings were recorded independently of the medical diagnoses (i.e., US findings did not automatically lead to medical diagnoses). We have specifically recorded all clinical findings exactly as they were in the medical records in order to also report possible discrepancies between individual clinicopathological and US findings and the final clinical suspicion of pancreatitis—yes or no.

Radiology reports were screened, and it was recorded if cats had a US diagnosis of pancreatitis (yes/no), referred to as USDx. The presence of the following pancreatic US findings was recorded: pancreatic enlargement, hypoechogenicity, hyperechogenicity, mixed echogenicity, hyperechoic mesentery, and peripancreatic fluid. The presence of the following hepatobiliary US findings was extracted from radiology reports: gallbladder sludge, hepatomegaly, hepatic hypoechogenicity, hepatic hyperechogenicity, and mixed echogenicity. Finally, the presence or absence of the following gastrointestinal US findings were extracted from radiology reports: lamina muscularis thickening, mesenteric lymphadenopathy, and gastrointestinal masses. All US examinations were performed by board-certified radiologists or residents supervised by board-certified radiologists.

Lipase activity was measured using the LIPC assay (LIPC, Roche on Cobas, Roche Diagnostics, Rotkreuz, Switzerland) with DGGR as the substrate [8]. Recorded hematologic variables (Sysmex XN-1000, Sysmex Swiss AG, Horgen, Switzerland) consisted of hematocrit, leukocytes, platelets, and segmented and band neutrophils and lymphocytes. Recorded serum biochemistry variables (Cobas c501, Roche Diagnostics, Basel, Switzerland) consisted of alkaline phosphatase activity, aspartate aminotransferase (AST) activity, alanine aminotransferase (ALT) activity, and concentrations of glucose, total protein, albumin, bilirubin, creatinine, urea, triglycerides (TG), cholesterol, and calcium.

### 2.2. Statistical Analyses

All statistical procedures were performed with SAS 9.4 (SAS Institute Inc.) and the individual cat was the smallest experimental unit. Age of cats, body weights of cats, as well as laboratory parameters, were analyzed in a general linear model with the procedure GLM, with the fixed factors of “group”, “sex”, and “group*sex”. The Student–Newman–Keuls test was applied as a post hoc test. The type I error threshold was defined as *p* ≤ 0.05, and data are presented as means with 95% confidence limits (Cls), (Cl 2.5%, 97.5%). Fisher’s exact test was applied to compare frequencies between groups (PD, NP) concerning the presence of clinical signs, comorbidities, and US findings. The type I error threshold for multiple comparisons of said frequencies was adapted by Bonferroni correction to *p* ≤ 0.016. Fisher’s exact test was also used to compare lipase activity between cats with and without a USDx within the 3 groups—PA, PD, and NP. Spearman’s rank correlation coefficients (rs) were used to assess correlations between lipase activity, serum urea, and serum creatinine concentrations in groups PA, PD, and NP.

## 3. Results

A total of 563 cats met the inclusion criteria. Pancreatitis alone (PA) was found in 33 cats (6%), 159 cats (28%) had a pancreatitis diagnosis and at least one additional concurrent disease (PD group), and 371 cats (66%) had no pancreatitis diagnosis recorded (NP).

**Figure 1:**
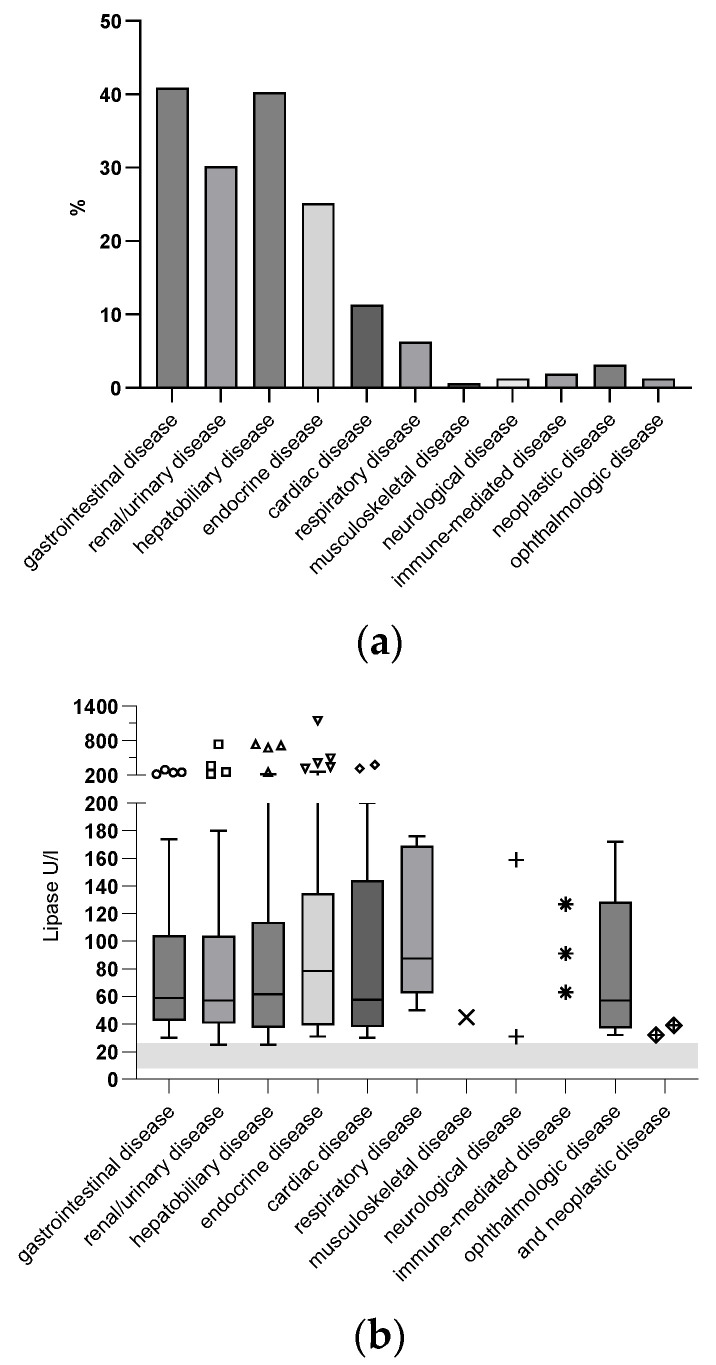
(**a**) Prevalence of comorbidities categorized by organ systems in the PD group. (**b**) Lipase activities associated with disease categories in the PD group. Box plots span the range from 25th to 75th percentile (IQR), whiskers mark minimum and maximum values excluding outliers which are represented separately as symbols. RI for lipase activity is indicated as a gray-shaded area.

During the same period, a total of 3998 cats were hospitalized with available lipase activity measurements. Compared with this denominator, a prevalence of 0.8% (33/3998) PA and 4% (159/3998) PD was found, while 14% (563/3998) of cats had a lipase activity > 30 U/L. Mean (95% confidence limit (Cl)) age for PA cats was 11.4 years (9.67, 13.1), for PD 12 years (11.4, 12.6), and 11.5 years (10.9, 12.0) for NP. Mean (Cl) weight for PA cats was 4.77 kg (4.34, 5.21) for PD 4.76 kg (4.51, 5.01) and 4.57 kg (4.34, 4.80) for NP. Age and body weight did not differ between groups. Mean (Cl) duration of clinical signs before presentation did not differ between groups, and was 11 days (−0.35, 22.5) for PA, 18.4 days (9.31, 27.4) for PD, and 28.7 days (17.7, 39.6) for NP. Mean (Cl) duration of hospitalization did not differ between groups either: PA 2.79 (2.29, 3.29), PD 3.59 (3.12, 4.06), and NP 3.23 (2.93, 3.53). Comorbidity prevalence summarized per category can be seen in Figure 1a.

Seventy-eight PD cats (49%) had one additional disease, 65 (41%) cats had two additional diseases, 15 (9%) cats had three additional diseases, and 1 (1%) cat had five additional diseases. Individual disease categories and distribution of lipase activities among groups is shown in Figure 1b.

**Figure 2:**
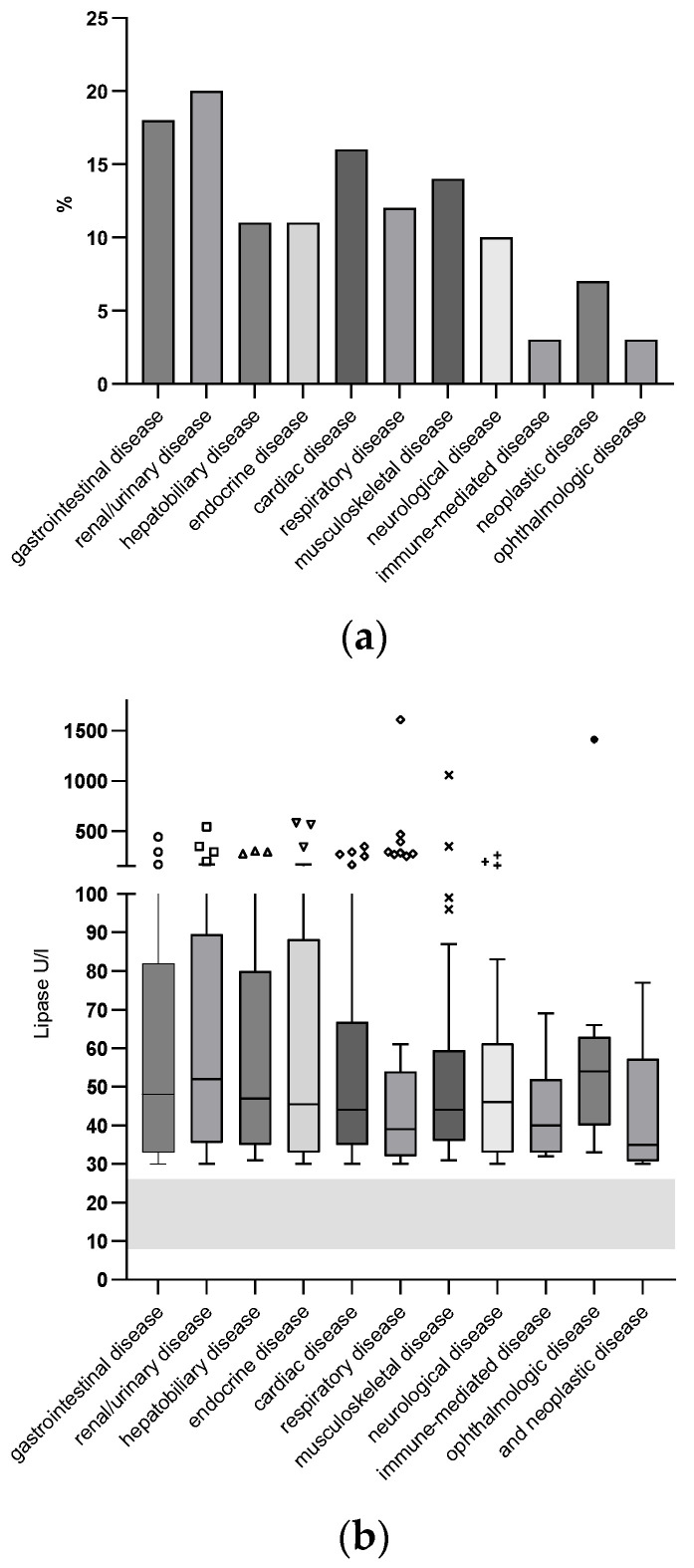
(**a**) Prevalence of comorbidities categorized by organ systems in the NP group. (**b**) Lipase activities associated with disease categories in the NP group. Box plots span the range from 25th to 75th percentile (IQR), whiskers mark minimum and maximum values excluding outliers which are represented separately as symbols. RI for lipase activity is indicated as a gray-shaded area.

Prevalence of disease categories and distribution of lipase activities in the NP group can be found in Figure 2a,b.

The number of cases for each group in PD and NP is illustrated in Figure S1 in the supplemental data.

### 3.1. Comparison of Clinical Signs between Groups

Anorexia (26/33, 79%), lethargy (24/33, 73%), and vomiting (17/33, 52%) were most frequently noted in PA cats. A similar pattern was observed in the PD and NP groups (Table 1). Anorexia and vomiting were significantly more common in PA cats compared to NP cats. Presence of a painful abdomen was uncommon overall (7/33 (21%) PA, 29/159 (18%) PD, 26/371 (6%) NP), but significantly more common in PA cats compared to NP cats. Pu/Pd was significantly more common in the PD group compared to PA cats. Diarrhea was uncommon, and bloody vomiting/diarrhea was very rare in all groups (Table 1).

### 3.2. Comparison of Laboratory Results between Cats with PA, PD, and NP

Mean (Cl) lipase activities did not differ significantly between cats (*p* = 0.27) in the PA (133 U/L, (79.0, 187)), PD (115 U/L, (92.1, 137)) and NP groups (80.1 U/L, (65.9, 94.3)). Minimal lipase activity increases between 30 and 40 U/L were found in 157/371 (42%) NP cats. Median lipase activity of 55 NP cats with a USDx was 57 U/L (IQR, 38–95 U/L). Lipase activities of cats with and without a USDx within the three groups did not differ significantly (*p* = 0.61; NP, *p* = 0.62; PA, *p* = 0.44). Lipase activities neither correlated significantly with the duration of disease before presentation when assessed for all cats with known disease duration (*n* = 480) nor when assessed per group.

Of all the laboratory variables, only ALT activity (RI, 20–93 U/L) (*p* = 0.005) and TG concentrations (RI, 0.4–1.5 mmol/L) differed between groups (*p* = 0.003). For both, the post hoc test did not find a difference between groups. Mean (Cl) ALT activity in NP cats was 219 U/L (147, 291), 168 U/L (106, 230) in PD cats, and 51.5 U/L (32.3, 70.6) in the PA group. AST activities exhibited a similar trend (*p* = 0.07). Mean (Cl) TG concentration in PA cats was 2.94 mmol/L (0.65, 5.23), 2.38 mmol/L (1.31, 3.46) in PD cats, and 1.39 mmol/L (0.89, 1.88) in the NP group.

Azotemia defined as serum creatinine concentration above the upper RI (163 μmol/L) was present in 4 (12%) PA cats, 49 (31%) PD cats and 143 (45%) NP cats, with a mean (Cl) serum creatinine concentration of 125 μmol/L (115, 136) in PA cats, 183 μmol/L (152, 245) in PD cats, and 236 μmol/L (207, 264) in NP cats. Creatinine concentration did not correlate significantly with lipase activities in cats in the PD (*n* = 170) or PA (*n* = 30) group, while a weak significant correlation (r_s_ = 0.123, *p* = 0.0188) was found in the NP group (*n* = 362). When assessed for all cats with lipase and creatinine measurements (*n* = 562), no significant correlation was found. Similarly, serum urea concentration did not correlate significantly with lipase activities in PD (*n* = 170) and PA (*n* = 30) cats, while there was a weak significant correlation (r_s_ = 0.168, *p* = 0.0014) with lipase activities in the NP group (*n* = 362). No correlation was found for any of the 562 cats with urea and lipase activity measurements.

### 3.3. US Findings Compared between Groups

**Figure 3:**
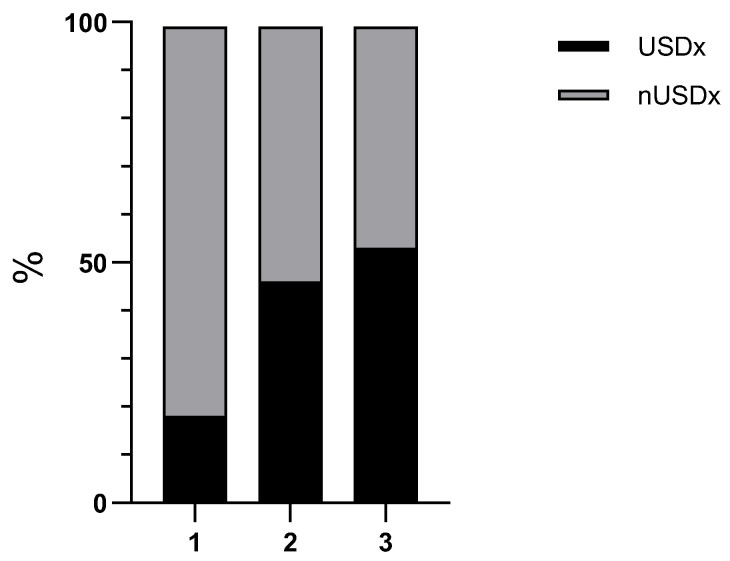
Bar diagram showing frequencies of an ultrasonographic diagnosis of pancreatitis (USDx) among the 3 groups (1 = NP, 2 = PA, 3 = PD). Cats with PA and PD had a USDx significantly more often compared to NP (*p* < 0.0001). The difference between PA and PD cats was not significant (*p* = 0.56).

Abdominal US was performed in 27/33 (82%) PA cats, 134/159 (84%) PD cats, and 157/371 (42%) NP cats. A USDx was present in 52% (15/33) of PA cats, 53% (85/159) of PD cats, and 15% (55/371) and NP cats, with a significant difference (*p* < 0.0001) between cats with pancreatitis (PA, PD) and cats without pancreatitis (NP) (Figure 3). A weak significant correlation (r_s_ = 0.129, *p* = 0.02) was found between a USDx and lipase activity when assessed for all 318 cats that had an abdominal US examination performed.

Pancreatic US findings for the three groups are summarized in Table 2.

Pancreatic enlargement, hypoechogenicity, and hyperechoic mesentery were significantly more common in cats with pancreatitis (PA, PD) compared to NP cats. A mixed-echo or hyperechogenic pancreas was less commonly found, and there were no differences between groups. Peripancreatic fluid was rarely observed. None of the hepatobiliary US variables was significantly different between groups. A hyperechoic liver was the most common (45% (62/138) PD, 39% (71/182) NP, 27% (7/26) PA) and hepatomegaly the second-most common hepatobiliary US abnormality (40% (55/138) PD, 35% (64/182) NP, 12% (3/26) PA). The difference between groups (*p* = 0.02) was not significant after Bonferroni corrections were applied. Similarly, no significant difference was found between groups when gastrointestinal US variables were compared. Lamina muscularis thickening was most common, with 48% (63/131) in PD cats, 34% (61/182) in NP cats, and 30% (8/27) in PA cats. Only 4 (15%) PA cats had US abnormalities limited to the pancreas, while 19/27 (70%) PA cats also had US evidence of concurrent enteropathy and 14/27 (52%) had US evidence of concurrent hepatopathy.

## 4. Discussion

In this retrospective study, we examined how often hospitalized cats with increased lipase activities receive a clinical diagnosis of pancreatitis and how often cats with suspected pancreatitis have comorbidities. We found that pancreatitis is relatively commonly diagnosed in the studied cohort with 34% (192/563), but the majority of these cats have at least one other disease, while pancreatitis alone (33/563, 6%) is rarely found. Even though no additional medical diagnoses were listed for 33 PA cats, 23/33 (70%) cats still had US evidence of either intestinal or hepatobiliary disease, leaving only 10/33 (30%) PA cats without extra-pancreatic US abnormalities. In addition, to our surprise, we found that 66% (371/563) of cats with hyperlipasemia had no pancreatitis diagnosis, even though a subset of these cats (55/371, 15%) also had US evidence of pancreatitis. We want to emphasize that our specific aim was to show the results as they were at our hospital (i.e., what was documented in the cats’ medical files) and not to have data retrospectively reviewed by an external panel, but to report exactly any discrepancies. We are aware that the main limitation of the study is the lack of clarity as to which criteria individual clinicians ultimately used to make the diagnosis of pancreatitis or not. However, we think our results reflect real-life clinical scenarios, namely, how to deal with lipase (and also pancreatic ultrasound) results in sick cats. Despite this limitation, the finding remains that hospitalized cats very rarely appear to have pancreatitis alone, and this is an original finding of this study. With our results, we want to stimulate a discussion on this difficult topic because we assume that other institutions have similar results. We can only assume this because no comparable results have been published to date. In the following, we discuss the points that we consider important.

Because cats with pancreatitis are usually nonspecifically ill and one of our aims was to assess how often sick cats with increased lipase are diagnosed with non-pancreatic diseases, we did not use predefined clinical signs as inclusion criteria, in order to not miss any cases. As it is still unknown at which lipase cutoff values pancreatitis is present [15,24] we included all cats with minimally increased lipase activity results compared to our current RI (6–26 U/L). The 30 U/L cutoff was chosen, as this value is currently emerging as our approximate new upper RI limit (*n*= 37 healthy cats included so far). Also, this cutoff is comparable to the RI used at the University of Guelph for the same LIPC Roche assay assessed in 60 healthy cats (RI, 12–32 U/L) [10].

Since abdominal US examinations are typically not performed in cats with primary cardiac or respiratory disease and younger cats with lower urinary tract disease often only receive US examinations limited to the urinary tract, lacking pancreatic US examinations was not regarded as an exclusion criterion either. It is quite possible that more cats fell into the NP group, because fewer cats in this group had a US examination. However, our results are in agreement with the previous literature that pancreatic US findings do not correspond well with lipase values [9,23,24]. The discrepancy between lipase and ultrasound results may be due to the fact that not all parts of the pancreas were seen during US examinations, that cats may have been highly acutely ill and pancreatic changes were not yet visible on US, or that cats with chronic pancreatitis had altered pancreata on US, but no increase in lipase. In mildly acute pancreatopathies, it is also possible that there are simply insufficient differences between acoustic impedance of abnormal and normal pancreata to permit clinically applicable characterization of pancreatic tissue. In addition, it is yet unclear when and with which pancreatic findings radiologists finally formulate a US diagnosis of pancreatitis. A similar discrepancy has been shown when pancreatic US findings were compared to pancreatic histology results in cats [25].

Our study differs from previous studies that also reported comorbidities in cats with a clinical diagnosis of pancreatitis [12,21,22]. In these studies, cats were either included when fulfilling predefined clinical signs [12,21,22] or excluded when several predefined comorbidity categories were also present [21,22]. Moreover, cats were only included when more severely ill [21] or only a selection of possible comorbidities were reported [22]. Also, the largest study to date providing information on frequently found comorbidities in cats with pancreatitis did not specify how many cats had pancreatitis alone without comorbidities [12]. We consider this valuable information, and our results provide the first reference for clinicians. It can be challenging to understand what disease is responsible for presenting complaints in a cat with laboratory evidence of pancreatitis but also presence of extra-pancreatic disease(s), given that clinical signs of feline pancreatitis are notoriously unspecific, and above all the two diagnostic cornerstones, US and lipase, agree only poorly.

Common concurrent diseases in PD cats were gastrointestinal disease (34%), hepatobiliary disease (33%), renal/urinary disease (25%), and endocrine disease (21%), followed by cardiac disease (9%). With the study design used, it is not possible to say with certainty which disease was the primary problem. Any of the diseases listed above may lead to compromised pancreatic perfusion causing a secondary or reactive pancreatopathy. Reactive changes might even be more likely if unrecognized chronic pancreatitis is already present. Alternatively, pancreatitis and comorbidities could have existed independently of each other. In principle, this dilemma applies to all previous studies on pancreatitis in cats where comorbidities were not explicitly excluded [7,12,21,22]. Concurrent hepatobiliary and gastrointestinal diseases have been well described in cats with histological [5,17,18,19,20] or clinical [12,21,22] evidence of pancreatitis and were somehow expected. The anatomy of the feline gastrointestinal tract certainly plays a role here. The small intestine is shorter compared to dogs, there is a higher concentration of duodenal bacteria, and the pancreatic duct joins the common bile duct before entering the duodenal papilla [26]. This increases the risk of duodenal bacteria ascending into pancreas and liver, resulting in parenchymal inflammation [26]. The frequent coexistence of gastrointestinal, hepatobiliary, and pancreatic inflammatory disease (often termed triaditis) has not only been reported in sick cats but also in cats showing no clinical signs [20]. Intestinal histopathological lesions of cats with triaditis were significantly more severe compared to cats where intestinal lesions alone were present, suggesting that chronic enteritis might play a role in the development of triaditis [20]. Pancreatitis is also known to be a common comorbidity in cats with diabetes [11,27]. Subclinical chronic pancreatitis with the development of fibrosis is thought to be the main cause of diabetes in cats [27,28]. Pancreatitis was subclinical in almost all cats with newly diagnosed diabetes in one study, and its clinical relevance was questioned by the authors [28]. It is less clear whether preexisting diabetes can also trigger pancreatitis in cats. Experimentally, a 10-day hyperglycemic clamp led to significant increases in intrapancreatic neutrophils in healthy cats [29]. It is thus possible that chronic hyperglycemia may also play a role in the pathogenesis of pancreatitis in cats [29].

The high number of cats with concurrent renal disease very likely reflects the high prevalence of kidney disease and pancreatitis in older cats [30,31]. We think that it is less likely that reduced renal clearance contributed to raised lipase levels, as creatinine and urea concentrations did not correlate significantly with lipase activities in cats with pancreatitis and all cats, and only poorly when assessed for cats without suspected pancreatitis. Similar conclusions were drawn in a recent study on lipase activity in cats with chronic kidney disease [32]. Only a minimal statistical difference was found and not deemed clinically relevant [32]. This fits in with the fact that studies in dogs showed no significant effects of experimentally induced acute kidney injury [33] and chronic renal failure [34] on lipase activity and PLI either. ALT activity differed significantly between groups, and the post hoc test did not find a difference between groups. Mean ALT activities were higher in NP and PD cats compared to within-RI mean ALT activity in PA cats. We assume this is due to the frequent occurrence of hepatobiliary disease in the first two groups. In a previous study, liver enzyme activities were significantly higher in cats with chronic pancreatitis compared to cats with acute necrotizing pancreatitis [5]. The authors also suspected that this was due to the higher prevalence of concurrent hepatobiliary disease in cats with chronic pancreatitis [5].

We found that TG concentrations were also significantly different between groups. Similar to ALT activities, the post hoc test did not find differences between groups, but mean TG concentrations were higher in cats with a pancreatitis diagnosis (PA and PD groups) and within RI in cats without a pancreatitis diagnosis (NP). In humans, hyperlipidemia is associated with acute pancreatitis, both as a precipitant and as an associated epiphenomenon [35]. Similarly, hyperlipidemia has also been the most commonly reported biochemical abnormality in cats with pancreatitis [4]. However, prospective studies are needed to investigate this in more detail.

As expected, the most common clinical signs in cats with pancreatitis were lethargy and anorexia, followed by vomiting. Similar to previous studies [5,12], a painful abdomen was only recorded in 21% (7/33) of cats with PA and 18% (29/159) of PD cats. Overall, our retrospective study design reveals no pattern as to whether cats with PA present clinically differently from cats with multiple diseases. This is certainly due to the variety of additional diseases, but it also underlines that sick cats per se often present nonspecifically ill. Pu/Pd was more common in the PD group because more cats had concurrent diabetes mellitus in this group. In contrast to dogs with acute pancreatitis [36,37], diarrhea is uncommon (15%) in cats with PA. This is similar to what is reported in cats with clinically more severe pancreatitis [5,21]. Diarrhea was only slightly more common (33/159, 20%) in PD cats where gastrointestinal disease was the largest comorbidity. Our results suggest that cats with pancreatitis alone are clinically comparable to cats with pancreatitis and comorbidities, but a prospective study is needed to better assess this. Still, the similarity of clinical signs renders formulations like “clinical signs consistent with pancreatitis” in cats less useful [38].

One could assume that cats with PA are more likely to have more acute disease and cats with PD more likely to have chronic pancreatitis. However, neither duration of disease before presentation nor length of hospitalization differed between groups. Also, PA cats are not significantly younger than PD cats and do not have higher lipases or more frequent US findings typically associated with acute pancreatitis, such as a hypoechogenic or enlarged pancreas or a hyperechoic mesentery. Our lack of differences between PA and PD is similar to lacking differences between acute and chronic pancreatitis when assessed by the diagnostic gold-standard histopathology [5].

To our surprise, lipase activities were not significantly different between groups. Indeed, quite high lipase activities were found in a subset of NP cats. We propose two possible explanations for this. We assume attending clinicians might not have listed an extra diagnosis of pancreatitis if a disease explaining all clinicopathological findings was present and no separate treatment was considered necessary for concurrent pancreatitis. This is currently only a theory, as the different assessment of different findings by clinicians was not investigated in this study. Increased lipase activities might have been regarded in the context of pancreatic hypoperfusion leading to secondary pancreatic damage without assuming that it reflected a primary pancreatopathy (i.e., collateral damage). This is supported by a significantly lower percentage of USDx in NP cats. However, 15% (55/371) of NP cats still had a USDx. Supplementary Table S1 gives an overview of the disease spectrum of these cats. All cats were severely ill, and frequent diagnoses were hepatobiliary disease, neoplasia, chronic kidney disease, and chronic enteropathy. Most likely, these cats also had pancreatitis, but severe extra-pancreatic disease seemed to have predominated the clinical picture, and thus a diagnosis of pancreatitis might not have been listed separately. Our results illustrate the limitation of our study well, namely, that we took the diagnoses from the medical histories. However, at the same time, this reveals an interesting aspect that has so far been described mainly in dogs: hyperlipasemia has been frequently found in critically ill dogs with a variety of diseases [39]. Pancreatic hypoperfusion was also a major point of discussion in that study [39].

The second explanation could be the presence of subclinical chronic pancreatitis in PD cats [1,2,14]. The literature emphasizes that older cats frequently have chronic pancreatitis, and thus increased lipase activities may have been attributed to chronic pancreatitis, which was not thought to be associated with the primary disease. Currently, little is known about the magnitude of lipase increases in cats with chronic pancreatitis. Lipase activities and fPLI concentrations in the range of the mean values of the NP group and higher have been described recently in clinically normal cats [15,40], as well as in sick cats without clinical suspicion of pancreatitis [13], supporting this explanation. Lipase activity and fPLI values in the range of 14–52 U/L and 2.3–15.7 μg/L have recently been reported in clinically healthy cats [40], in one case fluctuating between 35–118 U/L and 11–43 μg/L over the span of 1 year [40]. As mentioned above, the fact that in our hospital lipase activity is immediately available together with the routine clinical chemistry profile results without the need for an additional external request and thus without causing additional cost certainly plays a role here.

A major problem in the diagnosis of feline pancreatitis, and therefore also for this study, is that it is unknown at what lipase level pancreatitis is present. In humans, lipase activities threefold the upper RI limit are usually considered suspicious for pancreatitis. Perhaps this is similar in cats. At present, it is only known what lipase activities and fPLI concentrations are found in clinically healthy cats. However, ages of published reference populations have not been specified [8,10,15], and it is conceivable that older clinically healthy cats may have even higher lipase levels due to unrecognized mild chronic pancreatitis. Similar to our preliminary slightly higher lipase activity RI results, the fPLI RI has recently been corrected upwards to “capture more clinically relevant pancreatitis”, although it is still unclear exactly what this means [15]. This new provisional fPLI cutoff for diagnosing pancreatitis was based on calculations with an expected maximal specificity, but was not compared to a diagnostic gold standard [15], presumably because there is no established diagnostic gold standard for the definitive diagnosis of pancreatitis in cats apart from histology. Results of a recent study illustrate these diagnostic difficulties [24]: 71% (32) of 45 cats with a clinical diagnosis of pancreatitis had fPLI concentrations well within (the previous lower) RI; the median fPLI concentration of 45 cats with suspected pancreatitis was 1.8 mcg/L [24].

Concern has recently been raised that extra-pancreatic lipases may also hydrolyze the substrate DGGR in healthy cats and thus contribute to lipase activity in healthy cats without pancreatitis [41]. In that study, six healthy neutered cats were given heparin IV and lipase activities (Diazyme Laboratories, Inc., Gregg, CA, USA) were measured 10, 20, 30, 60, and 120 min after IV heparin administration [41]. Only one significant increase in lipase activities at 10 min post IV heparin compared with baseline was found. Because no RI for the lipase activity assay was given in that study, it remains unclear whether all results were also within RI [41]. We cannot fully rule out influences of extra-pancreatic lipases on our lipase assay, but believe that this is of minor concern, as higher variation between lipase activity and fPLI seems to happen mainly in healthy cats with low values within RI [40] compared to very strong correlations between both lipase assays in cats with suspicion of pancreatitis [2,9,11].

We specifically aimed to present all clinical findings and diagnoses as they were and not to retrospectively modify patient diagnoses based on study classification schemes by experts that were not involved when cats were hospitalized, as done in other studies [7]. We chose this approach because we think our findings illustrate a clinically relevant problem, namely, that hyperlipasemia reflecting some form of pancreatic injury is commonly associated with several different conditions and diseases in cats also, similar to what is described in dogs. This concept of a “secondary pancreatic injury” not necessarily reflecting primary pancreatitis has recently been discussed in cats [12,14]. In dogs, a large number of extra-pancreatic diseases have been described that are associated with increased lipase without a primary suspicion of pancreatitis, ranging from endocrine to infectious to cardiac to neurologic diseases. [42,43,44,45,46,47,48,49,50,51,52,53]. In cats, increased fPLI concentrations have also been reported in 8/80 (10%) cats where pancreatitis was considered unlikely [7]. Probably fewer cats with high fPLI values without suspicion of pancreatitis compared to our results were included in that study because external fPLI measurement had to be requested separately, in contrast to our study, where lipase activity was already included in the routine biochemistry profile [7].

Our study has several limitations mostly related to the retrospective study design. Importantly, we do not know which criteria were ultimately used to formulate a diagnosis of pancreatitis or why in such cases increased lipase activities and/or US findings did not lead to a clinical diagnosis of pancreatitis. In principle, this limitation also applies to radiological diagnoses, as we do not know when radiologists ultimately formulate a formulate an ultrasonographic diagnosis of pancreatitis based on the combination and severity of pancreatic US findings. Moreover, not all data points were available for all cats. Measurement of acute-phase proteins such as serum amyloid A concentration (SAA) would have been helpful to differentiate acute from chronic pancreatitis. Unfortunately, SAA was not yet part of the biochemical profile in our hospital at the time of data collection. To obtain a more complete picture of lipase levels in cats with and without a diagnosis of pancreatitis, it would have been ideal to include cats with normal lipase results also. However, this was not possible due to the large number of cases. Because of the comparatively low sample size of the PA group versus both other groups, frequency tabulations regarding the occurrence of clinicopathological and imaging changes in this group must be interpreted cautiously due to limited statistical confidence in estimation. Because of our main limitation, the likely different prioritization of pancreatic findings by different clinicians, a pancreatitis diagnosis-independent analysis using only the level of lipase activities in a cluster analysis, appears to be a promising way to investigate the correlation between clinical, laboratory, and imaging findings in a diagnosis-independent manner.

## 5. Conclusions

In summary, we show that pancreatitis alone is rare in cats. Most cats present with multiple diseases, and many sick cats with a variety of non-pancreatic diseases also have increased lipase activities. Prospective studies are needed to investigate whether this reflects a reactive pancreatopathy or the presence of concurrent clinical or subclinical chronic pancreatitis.

## Figures and Tables

**Table 1:** Presence of clinical signs among cats in the PA, PD, and NP groups.

Clinical Signs	PA (*n* = 33) *n* (%)	PD (*n* = 159) *n* (%)	NP (*n* = 371) *n* (%)	*p*-Value
Anorexia	26 (79) ^bc^	107 (67) ^b^	159 (43) ^a^	<0.0001
Lethargy	24 (73) ^bc^	93 (58) ^b^	184 (50) ^ab^	0.01
Vomiting	17 (52) ^ab^	90 (57) ^b^	119 (32) ^a^	<0.0001
Painful abdomen	7 (21) ^ac^	29 (18) ^a^	26 (6) ^b^	0.0001
Diarrhea	5 (15) ^a^	33 (21) ^a^	47 (13) ^a^	0.06
Weight loss	4 (12) ^a^	48 (30) ^a^	78 (21) ^a^	0.02
Polyuria and polydipsia	1 (3) ^bc^	37 (23) ^a^	52 (14) ^b^	0.003
Bloody vomiting	0 (0) ^a^	5 (3) ^a^	4 (1) ^a^	0.21
Bloody diarrhea	0 (0) ^a^	5 (3) ^a^	4 (1) ^a^	0.21

Note: Fisher’s exact test was applied to test for differences in frequencies of clinical signs between groups. Due to multiple comparisons between single groups, the Bonferroni correction was applied to adjust the type I error threshold to *p* ≤ 0.016. Frequencies between groups not sharing a common superscript (a,b,c) are significant at *p* ≤ 0.016. Pancreatitis alone (PA), pancreatitis with concurrent disease (PD), no clinical diagnosis of pancreatitis (NP).

**Table 2:** Frequencies of US findings among groups.

US Findings Pancreas	PA (*n* = 27) *n* (%)	PD (*n* = 134) *n* (%)	NP(*n* = 157) *n* (%)	*p*-Value
Enlargement	16 (59) ^ac^	70 (52) ^a^	37 (24) ^b^	<0.0001
Hypoechogenicity	12 (44) ^ab^	60 (45) ^a^	44 (28) ^b^	0.008
Hyperechoic mesentery	12 (44) ^ac^	48 (36) ^a^	26 (17) ^b^	<0.0001
Mixed echogenicity	10 (37) ^a^	43 (32) ^a^	36 (23) ^a^	0.11
Hyperechogenicity	8 (30) ^a^	24 (18) ^a^	18 (11) ^a^	0.04
Fluid peripancreatic	4 (15) ^a^	11 (8) ^a^	12 (8) ^a^	0.46

Note: Fisher’s exact test was applied to test for differences in frequencies of ultrasonographic findings between groups. Due to multiple comparisons between single groups, the Bonferroni correction was applied to adjust the type I error threshold to *p* ≤ 0.016. Frequencies between groups not sharing a common superscript are significantly different at *p* ≤ 0.016.

## Data Availability

Data available on request due to restrictions, e.g., privacy or ethical reasons. As the study is retrospective, no owner consent was signed, and personal data is therefore not publicly available.

## Supplementary Materials

The following supporting information can be downloaded at: https://www.mdpi.com/article/10.3390/ani14101479/s1, Figure S1: Number of cats with different disease categories in PD (left) and NP groups (right). (PD, pancreatitis with concurrent diseases; NP, no clinical diagnosis of pancreatitis). Table S1: Disease spectrum of 55 NP cats with an USDx (NP, no clinical diagnosis of pancreatitis; USDx, ultrasonographic diagnosis of pancreatitis; EHBDO, extrahepatic bile duct obstruction).
