# Supplementary material for: Evaluation of Disease Spectrum in Hospitalized Cats with Hyperlipasemia: Pancreatitis Alone Is Rare, Hyperlipasemia without Suspected Pancreatitis Is Common
Source: Animals (Basel). 2024 May 16;14(10):1479. doi: 10.3390/ani14101479 (PMC11117254; doi:10.3390/ani14101479)
Supplement: Supplementary file 1 [file animals-14-01479-s001.zip › animals-2982561-supplementary.pdf]

Supplemental data

Figure S1: Number of cats with different disease categories in PD (left) and NP groups (right). (PD, pancreatitis with concurrent diseases; NP, no clinical diagnosis of pancreatitis)

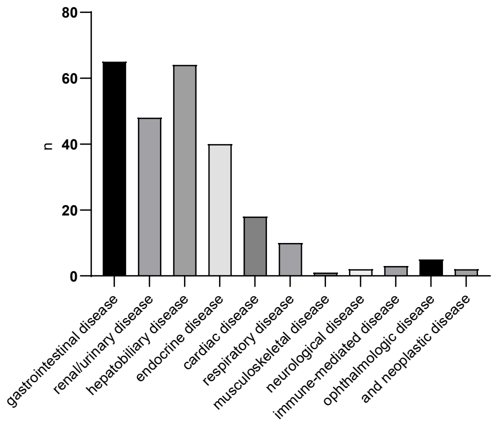

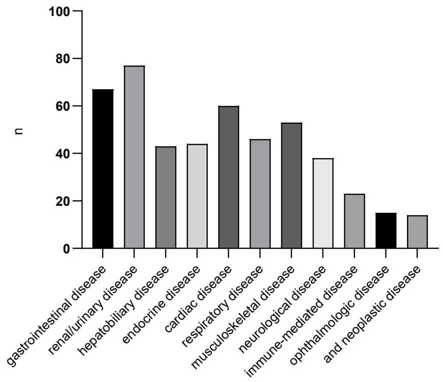

Table S1: Disease spectrum of 55 NP cats with an USDx (NP, no clinical diagnosis of pancreatitis; USDx, ultrasonographic diagnosis of pancreatitis; EHBDO, extrahepatic bile duct obstruction).

Disease	Number
Hepatobiliary disease (incl. EHBDO n= 6)	10
Neoplasia	9
Chronic kidney disease	8
Chronic enteropathy	8
Diabetic ketoacidosis	7
Cardiac disease	5
Diabetes mellitus	5
CNS disease	3
Anemia	3
Hepatic lipidosis	2
Acute kidney disease	2
Respiratory disease	2
Lower urinary tract disease	2
Subcutaneous ureteral bypass	1
Polyarthritis	1
Intestinal foreign body	1
Sepsis	1
Anaphylaxis	1
